# Spurious Autobiographical Memory of Psychosis: A Mechanistic Hypothesis for the Resolution, Persistence, and Recurrence of Positive Symptoms in Psychotic Disorders

**DOI:** 10.3390/brainsci13071069

**Published:** 2023-07-13

**Authors:** Eric Y. H. Chen, Stephanie M. Y. Wong, Eric Y. H. Tang, Lauren K. S. Lei, Yi-nam Suen, Christy L. M. Hui

**Affiliations:** 1 Department of Psychiatry, School of Clinical Medicine, LKS Faculty of Medicine, The University of Hong Kong, Hong Kong, China; steph.my.wong@gmail.com (S.M.Y.W.); erictanghku@gmail.com (E.Y.H.T.); kashun.lei@gmail.com (L.K.S.L.); suenyn@hku.hk (Y.-n.S.); christy@lmhui.com (C.L.M.H.); 2 The State Key Laboratory of Brain and Cognitive Sciences, The University of Hong Kong, Hong Kong, China

**Keywords:** psychotic disorders, relapse, treatment response, salience, memory, dopamine, hippocampus

## Abstract

Psychotic disorders are complex disorders with multiple etiologies. While increased dopamine synthesis capacity has been proposed to underlie psychotic episodes, dopamine-independent processes are also involved (less responsive to dopamine receptor-blocking medications). The underlying mechanism(s) of the reduction in antipsychotic responsiveness over time, especially after repeated relapses, remain unclear. Despite the consistent evidence of dopamine overactivity and hippocampal volume loss in schizophrenia, few accounts have been provided based on the interactive effect of dopamine on hippocampal synapse plasticity mediating autobiographical memory processes. The present hypothesis builds upon previous works showing the potential effects of dopamine overactivity on hippocampal-mediated neuroplasticity underlying autobiographical memory, alongside known patterns of autobiographical memory dysfunction in psychosis. We propose that spurious autobiographical memory of psychosis (SAMP) produced during active psychosis may be a key mechanism mediating relapses and treatment non-responsiveness. In a hyperdopaminergic state, SAMP is expected to be generated at an increased rate during active psychosis. Similar to other memories, it will undergo assimilation, accommodation, and extinction processes. However, if SAMP fails to integrate with existing memory, a discontinuity in autobiographical memory may result. Inadequate exposure to normalizing experiences and hyposalience due to overmedication or negative symptoms may also impede the resolution of SAMP. Residual SAMP is hypothesized to increase the propensity for relapse and treatment non-responsiveness. Based on recent findings on the role of dopamine in facilitating hippocampal synapse plasticity and autobiographical memory formation, the SAMP hypothesis is consistent with clinical observations of DUP effects, including the repetition of contents in psychotic relapses as well as the emergence of treatment non-responsiveness after repeated relapses. Clinical implications of the hypothesis highlight the importance of minimizing active psychosis, integrating psychosis memory, avoiding over-medication, and fostering normalizing experiences.

## 1. Background

Psychotic disorders such as schizophrenia include some of the most debilitating conditions and substantially contribute to the global burden of disease [1,2]. They encompass a group of conditions that, despite being characterized by some heterogeneous phenomenological features, may share common pathogenic mechanisms that lead to the expression of psychotic episodes [3,4,5]. In particular, their relapsing course and progressive emergence of treatment non-responsiveness appear to be present across psychosis spectrum disorders [6,7,8,9]. Despite their mechanisms not being fully understood, the prevention of relapse and treatment refractoriness in psychosis remain important challenges impacting the long-term outcome of psychotic disorders.

One of the most widely studied theories of psychosis and its recurrence is the dopamine overactivity hypothesis [10,11]. An overactive dopamine system has been construed as the final common pathway to psychotic episodes [12]. Nevertheless, there is evidence that the manifestation of psychosis also involves dopamine-independent processes at multiple etiological levels [13], such as other neurotransmitter systems (e.g., GABA, glutamate, or cannabinoid receptors), brain connectivity changes [14], as well as psychological schema (such as persecutory and paranoid schemas) [15,16,17,18,19], which may link to childhood adversity and adult life events [20,21,22,23]. Of note, such non-dopamine processes may appear not only at the time of illness onset but may also emerge later during the progression of the illness [24]. The longitudinal evolution of these factors could contribute to the propensity to relapse and diminishing responsiveness to medication [9,25]. The roles of possible non-dopaminergic mechanisms in relapse and refractory psychosis have been intensively studied, but understanding of the underlying pathways remains limited [26,27].

Importantly, the longitudinal course of psychotic symptoms cannot be considered in isolation from the incremental and cumulative retention of information in the brain over time. This involves the study of memory and neuroplasticity. While changes in memory have been extensively studied in psychotic disorders, they have often been studied in terms of deficits in memory performance [28,29]. Few studies have systematically explored the possible impacts of the memory traces that were formed during the psychotic episode. We propose a novel perspective in which individualized past autobiographical memories of psychotic experiences are hypothesized to play a key role in priming one’s future propensity for psychotic experiences. It is proposed that consolidated autobiographical memory of psychotic experiences contributes to a dopamine-independent process driving relapses and treatment non-responsiveness. Accordingly, memory traces related to experiences *during* psychotic episodes (“spurious autobiographical memory of psychosis”) are considered to be encoded in the brain and not only persist after remission but would also play a critical role in determining the future risks for relapse and treatment response. 

During the relapse of a psychotic episode, interpretations of the environment with excessive self-reference and threat have been one of the key factors [30,31]. These interpretations depend not only on incoming perceptual information (bottom-up processes) but also on top-down processes involving pattern completion based on information retrieved from memory (e.g., past schemas) [32,33]. Top-down processes provide a mechanism whereby past autobiographical memories of psychosis could prime the retrieval of self-referential and threat schema. As such, persistent past memories of psychosis could increase the propensity towards self-referential and threat-based interpretations in the future, thereby increasing the chance of a re-experience of psychosis symptoms [34,35].

We continue this article with a review of recent knowledge on memory processes in psychotic disorders, followed by an outline of the key features of the proposed *spurious autobiographical memory of psychosis* (SAMP) hypothesis. We then discuss the neurobiological underpinnings of SAMP and compare them with the experience of spurious memories in other related conditions, including substance-induced psychosis and post-traumatic stress disorder (PTSD). Clinical implications and future directions of the SAMP hypothesis are discussed. 

### Memory Processes in Psychosis

Memory can be broadly divided into explicit and implicit memories. Explicit memory includes the registration and recall of short-term retention of information (episodic memory), long-term personalized real-world experiences (autobiographical memory), as well as general knowledge of the world (semantic memory). In contrast, implicit memory includes learned associations between perception, action, and emotion that involve information that may not have been consciously experienced and is more difficult to express via language [36,37]. Episodic memory is the process of laying down memories about day-to-day personal, real-life experiences [38]. Learned associations in episodic memory can be transferred across situations and become part of semantic memory, which can be formed when the recurrent regularity of the knowable world acquires a representation of independent personal events [39]. Clusters of such information may be used to construct high-level representations (sometimes described as schemas), which provide top-down contexts to guide the interpretation of low-level perceptual information [38]. 

As suggested by Jackson, different neurocognitive processes, such as memory, are mediated by a hierarchical structure in the brain where higher-level processes influence the functions of lower-level processes [40]. This hierarchical structure is consistent with the model of brain functions proposed by Luria [41] (i.e., the sensory-perceptual unit, the motor unit, and the higher cortical unit) and supports the idea that memory processes involve a complex interplay between different brain subsystems. More recent observations suggest that the laying down of perceptual and affective information in new experiences is mediated by the lower-level amygdala and hippocampus [42,43], while the integration of new information into existing knowledge for updating schemas is supported by the higher-level prefrontal cortex [44]. These reported interactions between the ventromedial prefrontal cortex and the hippocampus provided additional support for the theory that schemas modulate lower-level perceptual processing, with the two involved regions either in synchrony or competition [45]. 

Memories of new experiences are “integrated” with existing experiences through processes of assimilation and accommodation [46]. Those that fit in closely with prior experiences are “assimilated” into existing memory schemas as exemplars of similar events. Meanwhile, experiences that are significantly distinct from existing memories require a revision of the existing memory frameworks (schemas) before they can be coherently integrated (a process of “accommodation”). 

Explicit memory is mainly mediated by the hippocampal system (including the amygdala), while implicit memory is mediated by the striatal systems [47,48,49]. There is evidence that the capacity for explicit memory processes, such as working memory, short-term memory, episodic memory, autobiographical memory, and semantic memory, is impaired in psychotic disorders [50,51,52,53]. An increasing number of studies have also reported impairments in implicit memory in psychosis [52,54,55]. In addition, deficits in metamemory domains, such as confidence judgment, source memory, and event order, have also been reported [56,57,58,59]. 

However, most existing studies of memory in psychosis adopt a neuropsychological approach and focus on the impacts of reduced memory capacity for neutral stimuli [18,60,61], rather than *the formation and persistence* of anomalous memories. Most of these studies have been carried out in patients during a stable, non-psychotic state (i.e., when active psychotic episodes have been stabilized) [62]. As a result, the memory of individual patients *during acute psychosis* has been less thoroughly studied [50]. In particular, it remains unclear how memories are formed during active psychotic episodes, how they may be integrated with one’s prior pre-psychotic autobiographical memory, and how they may persist over time and affect future experiences of psychosis. 

## 2. The Spurious Autobiographical Memory of Psychosis (SAMP) Hypothesis

Based on recent observations of interactions between dopamine activity and autobiographical memory processes, we propose that spurious autobiographical memory of psychosis (SAMP) may provide a mechanistic account for relapses and treatment non-responsiveness. Key components of the SAMP hypothesis are outlined as follows:

1. Memory encoding and consolidation are facilitated by salience and mediated by the midbrain dopamine system.
2. These memory traces can be explicit or implicit, which are respectively mediated by the hippocampal and striatal systems; although the SAMP hypothesis focuses on the former, interactions between explicit and implicit memory systems are important.
3. During the hyperdopaminergic active psychotic state, the heightened registration and consolidation of experiences of psychosis (related to Hebbian processes at glutaminergic pyramidal cell synapses in the hippocampal CA3 and CA1 fields and facilitated by increased extracellular dopamine levels in the same synapses) are expected to result in spurious autobiographical memories that remain as long-term memory traces.
4. The contents of these memory traces are often incongruous with premorbid (or remission) memory traces. This results in difficulties in the assimilation and accommodation of memory with existing pre-psychotic memories.
5. The inability to integrate SAMP with premorbid/remission memory increases their chances of distinctive retrieval upon presentation of stimuli linked to the original psychotic episode, increasing the risks for future relapse.
6. While dopamine-blocking pharmacotherapy can prevent the formation of new spurious autobiographical memories, memory traces that have already been established cannot be erased and can only be replaced by “normal” experiences in a process of extinction, which can happen in remission but at a much slower rate. The speed of normalization is further reduced if there is hyposalience due to a hypodopaminergic state (e.g., negative symptoms or neuroleptic-induced deficit syndrome).
7. The risks of relapse and treatment non-responsiveness are hypothesized to be related to the cumulative load of SAMP. SAMP may play an important role in mediating the relationship between “duration of active psychosis” and relapse.

### 2.1. Longitudinal Evolution of Autobiographical Memory Traces Related to Psychosis

**Figure 1:**
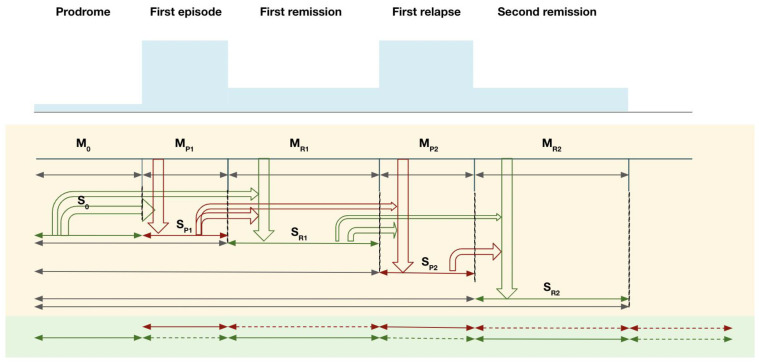
Spurious autobiographical memory of the psychosis model in different stages of a psychotic disorder: illustration of the relationship between psychosis memory and remission memory. Note: M = foreground autobiographical memory; S = background schema (integrated contextual memory).

The SAMP evolves in stages, from prodrome to subsequent psychotic episodes and their remissions. At each stage, memories of foreground experiences (memory M) are encoded and integrated with existing background memory contexts (schema S). Figure 1 illustrates the evolution of these processes with an example from the prodrome to the first and second psychotic episodes. 

In the prodromal stage, pre-psychotic experiences (M_0_) provide input into pre-psychotic schema (S_0_). During First-episode psychosis (FEP), new psychotic memories (M_P1_, red arrow) are encoded. In a hyper-salience condition during FEP, memories of psychotic life experiences are more likely to be consolidated, but their contents are more difficult to assimilate into S_0_, thus requiring more accommodation in the existing schema in order to integrate them coherently (accommodation mode, relatively larger changes in the schema S_0_). If this integration is incomplete, a newly formed psychosis memory schema (S_P1_) cluster is likely to persist. There are two possible outcomes of interactions between M_P_1 and S_0_ in the formation of S_P1_: (1) The new memories M_P1_ integrate well with S_0_ to build up S_P1_ (S_0_-M_P1_); (2) M_P1_ is very different from S_0_ and fails to integrate fully into S_0_, thereby generating a segregated S_P1_ (S_0_, S_P1_), resulting in two relatively distinct memory clusters S_0_ and S_P1_ (green and red). 

During remission, normalized new memories of post-psychotic remission experiences (M_R1_) are formed. M_R1_ integrates with M_0_ and S_0_ to form a remission schema (S_R1_) that is likely to align more with the previous non-psychotic cluster (S_0_) (i.e., the green cluster). If psychosis memory (S_P1_) is well integrated with S_R1_, normalization of psychosis memories takes place, allowing remission experience S_R1_ to interact with the memory formed in psychosis S_P1_ in such a way that normalized associations gradually replace the spurious associations in S_P1_ [63]. This process is expected to be less effective if S_P1_ is less well integrated with S_R1_. In this case, failure of integration may result in segregation of memory into two separate compartments: “spurious autobiographical memory of psychosis, SAMP”, and “non-spurious autobiographical memory”, with fewer connections between them [64]. In SAMP, “extinction” of spurious associations between stimuli may take place over longer time periods. As synaptic plasticity is already formed to represent SAMP traces, which do not automatically reverse when dopamine hyperactivity is regulated by antipsychotic blockage of dopamine receptors [65,66], coupled with the slower rate of learning in remission with normalized dopamine activities, spurious connections are slowly overwritten by repeated exposure to normal non-psychotic daily life experiences compared to their rapid formation during active psychotic states. Importantly, the fading of spurious memory would also be compromised if the normal detection of salience is reduced by a hyposalient state (e.g., with higher doses of anti-dopaminergic medication or when exposure to a range of experiences is reduced with social withdrawal as a result of negative symptoms) [12,67].

During relapse, memories (M_P2_) of the second-episode psychosis (SEP) experiences are formed. M_P2_ is laid down to form the second psychosis schema (S_P2_) that is more likely to be compatible with the psychotic S_P1_ (i.e., the red cluster) and separated from the non-psychotic S_R1_ (i.e., the green cluster). Failure of integration would result in the compartmentalization of psychotic and non-psychotic memory clusters. Spurious autobiographical memories of psychosis would accumulate longitudinally along the course of remissions and relapses.

### 2.2. Neurobiological Underpinnings of the Spurious Autobiographical Memory of Psychosis

#### 2.2.1. Dopamine and Hippocampal Function in Psychosis

The SAMP hypothesis proposes that abnormal dopamine-hippocampal regulation during psychosis is a core mechanistic process that intensively encodes and consolidates aberrant autobiographical memory traces, and that these memory traces play important roles in the longitudinal course of psychotic disorders. 

While the hippocampus has been considered one of the upstream regulators of midbrain dopamine function [68], the potential downstream effect of dopamine overactivity on hippocampal function has been less considered. For instance, intrinsic hippocampal dysfunction has been formulated as a mechanistic model for psychotic disorders [69]. Psychotic contents have also been pointed out as being plausibly consolidated via the CA subfields of the hippocampus into the normal declarative memory pathways. In addition, it has also been suggested that the role of dopamine-hippocampal interactions is not limited to schizophrenia but is rather applicable transdiagnostically to conditions involving a range of states with underlying dopamine dysregulation (e.g., [70,71]). However, the discussion in the literature to date has seldom incorporated the emerging evidence of dopamine interactions with hippocampal memory processes. 

Indeed, the mechanistic role of dopamine in implicit associative learning has been suggested in earlier studies on striatal synaptic plasticity [72,73]. Studies on implicit associative memory in psychosis (via learning paradigms such as latent inhibition, learned irrelevance, and blocking) have been taken to investigate the effects of dopamine on the striatum [74,75,76,77]. Notably, aberrant striatal connectivity has recently been linked to psychotic relapses [14]. 

More recently, in healthy subjects, the role of extracellular dopamine in the consolidation of autobiographical and emotional memory in the hippocampus and the amygdala has also been more actively explored [78,79]. These observations provide a coherent context for highlighting the interaction between dopamine and hippocampus functions and their roles in autobiographical memory in understanding psychotic disorders. 

#### 2.2.2. Dopamine-Mediated Salience and Hippocampal Interaction

Dopamine plays a key role in the SAMP hypothesis. In addition to its roles in reward, prediction error, salience, and novelty, emerging evidence suggests that dopamine modulates synapse modification in the hippocampus and regulates episodic memory in favor of salient novel events [80,81]. Specifically, the role of the CA3 subfield in the hippocampus has been identified as the site of memory formation for an autobiographical event, leading to the ability to re-experience the event through recall [82]. Functional imaging evidence confirms that midbrain dopamine projections to the hippocampus mediate human episodic/autobiographical memory [83,84] by directly facilitating long-term potentiation (LTP) in synapsis in the hippocampus [79]. This probably involves dopamine projections from the ventral tegmental area (VTA) and locus coeruleus (LC) to the dentate gyrus (DG)-CA3 microcircuit [85]. Importantly, like in real-life autobiographical memory, dopamine-dependent facilitation of neuroplasticity can be observed after a single episodic event [81]. Dopamine activation of D1/D5 receptors in the hippocampus facilitates the consolidation of new memory traces after they are encoded following glutaminergic activation of NMDA receptors [86]. 

Interestingly, in addition to VTA dopamine projections, noradrenaline projections from the LC to the DG also act to facilitate LTP in the hippocampus. In addition, memory consolidation has been further elaborated by the co-release of dopamine (with noradrenaline) from the LC to the hippocampus [87]. It has been proposed that novelty experiences preferentially activate the LC-CA3 projection, which would result in the accommodation of distinctive autobiographical memories of unfamiliar events. Meanwhile, experiences that share more similarities with past experiences would activate the (VTA)-hippocampal (CA1, CA3) projection, which would preferentially facilitate assimilation into past contextual experiences [87]. 

Recent investigations into memory encoding in healthy participants have suggested a complex process in which low-level sensory and affective components of a memory representation are distinguished from high-level contextual information [88]. Mildly negative experiences may strengthen low-level representation (amygdala-based, CA1, noradrenalin-related) while weakening high-level coherent, contextual, pattern completion associative encoding (CA3, hippocampal-based, dopamine-related) [89]. These observations suggest that on top of the general facilitation effect of increased salience, negative events may compromise the integration of coherent contextual memory in schema [90,91]. While these studies were carried out with mildly negative stimuli in healthy subjects, they suggest that emotions may both facilitate increased consolidation of part of the representation while also weakening the link with contextual information, thus potentially compromising the formation of integrated representations over time. 

#### 2.2.3. Increased Salience and Consolidation of Spurious Autobiographical Memory of Psychosis

As aforementioned, the perceived environment is monitored through ongoing comparison with predictions generated internally from past experiences and knowledge [92,93]. When deviation from the prediction is noted, a salience signal is generated in the brain, accompanied by a subjective sense that information of significance is present in the environment [94,95]. It has been proposed that salience detection is largely signaled by the dopamine system [30,96]. 

During psychotic episodes, aberrant dopamine overactivity and the associated heightened sense of salience accompany the experience of psychotic symptoms such as hallucinations and delusions [97,98]. These subjectively realistic experiences take place in clear consciousness and are expected to be registered as explicit autobiographical memories (similar to salient experiences in non-psychotic healthy states). Spurious associations between individual components of the memories are strengthened by Hebbian synaptic plasticity with the facilitation of an overactive dopaminergic state. 

The state of increased salience further facilitates the consolidation of not only explicit autobiographical but also implicit associative memory [75,81]. Spurious autobiographical memory of psychosis may therefore consist of both autobiographical memory of the core psychotic experience itself as well as indirect associations between incidental elements featured in the autobiographical memory, as well as implicit associations and emotional memories. While autobiographical memory may be accessible to subjective awareness, the latter may not be explicitly presented to the individual but constitute memory traces linked via associative processes—both of which can provide the basis for future retrieval [64]. 

### 2.3. The Fate of SAMP Traces and Their Failure of Integration

After the rapid encoding and consolidation of SAMP during the hyperdopaminergic active psychotic state, the subsequent fate of these memory traces during the post-psychotic state has not been fully investigated. While deficits in meta-memory and contextual memory have been observed in psychosis [99,100,101,102,103], psychotic experiences are generally reported as being recallable after a psychotic episode [104,105]. However, the extent to which one’s autobiographical memory of psychotic experiences can coherently integrate with prior life experiences during remission before and after a psychotic episode remains unknown. Studies of the memory of individualized psychotic experiences are seldom addressed in conventional neurocognitive studies of psychosis; instead, some relevant observations have been noted in the study of the coherence of autobiographical memory in psychotic disorders.

In the memories of one’s personal experiences, events across one’s life span are largely intertwined to generate a coherent autobiographical memory, which gives a sense of self-identity and continuity. Coherence consists of temporal, causal, and thematic aspects [106] and is attained by reflective “autobiographical arguments” of “self-defining memory”, in which “self-event” connections are key processes [105,107]. Autobiographical arguments have been viewed as important memory-based mechanisms that help individuals maintain a coherent sense of self and life experiences, particularly in the face of major life changes (e.g., migration, occupation change, major illness, significant loss), by connecting one’s experiences to the “self” [108]. Through such “self-event links”, episodic experiences are closely related to self-values, perspectives, and relationships [109]. 

As expected, autobiographical memory can be enhanced by salience and emotional links [110], as well as by thematic coherence [111]. When drastic life changes impose an “autobiographical discontinuity”, sense-making is sought to bridge the information by embedding the change in a more coherent and broadened narrative plot in order to integrate the disruptive event into one’s life narrative [112]. Recent studies suggest that a greater autobiographical discontinuity after experiences of major life stressors can increase intrusive memories and emotional hyperarousal [108,112]. Reduced biographical coherence and vividness of memories have recently been found to be associated with psychotic-like experiences in the general population [113].

#### Challenges in the Integration of Spurious Autobiographical Memory of Psychosis into Pre-Existing Non-Psychotic Autobiographical Memory

The disrupted experience of self and sense of agency are prominent features of psychosis. The encoded experience during active psychosis, therefore, understandably differs from the experiences encoded during non-psychotic states in some dimensions (e.g., in terms of self-relatedness and threats [32,114]). This presents challenges to the integration of autobiographical memory representations between psychotic and non-psychotic periods. 

A recent study has further found that different components of autobiographical memory are affected by psychotic-like experiences in the general population [113]. In particular, psychotic-like experiences were found to be associated with increased *involuntary* autobiographical memory recollection (reliving, rehearsal, centrality in personal life story, and emotional intensity), as well as reduced clarity of self-related information. This study offers some support to the view that the ability to relate events to the self in a coherent manner may be compromised by disrupted self-representations during psychotic experiences, thus presenting challenges to the integration of such spurious clusters of memory. Importantly, the formation of a cluster of memories related to the psychotic-like experience may be suggested by their being associated with not only more involuntary recall but also with a “broader enhanced autobiographical recollection of personal events, including reliving, mental imagery, content, and belief of what occurred” [113]. 

The failure to integrate specific episodic memory into a coherent context has also been linked to a relative increase in overgeneral autobiographical memory (OGM), i.e., general knowledge-based rather than personal event-based memories [104]. Studies of autobiographical memory in schizophrenia spectrum disorders have indeed reported significantly reduced memory specificity, vividness, clarity, and conscious recollection ability in patients [50]. These patterns of reduced specificity in episodic memory and increased OGM may represent transdiagnostic features of conditions in which autobiographical continuity is disrupted and may be applied to psychotic disorders as well as post-traumatic stress disorders and affective disorders [115,116]. Similarly, compared to the biographical disruption resulting from changes in real-life circumstances and traumatic experiences, those resulting from psychotic episodes may pose a unique challenge to autobiographical memory integration. Patients with schizophrenia have been found to produce more OGM and fewer self-defining memories [105]. The contents of these memories are also more related to life-threatening events as compared to healthy controls [117]. 

Notably, the temporal distribution of autobiographical memory in schizophrenia patients appears to show a characteristic pattern, wherein deficits in the memory of personal detail-rich autobiographical events are observed particularly around the onset of illness, suggesting a discontinuity of autobiographical memory for that period [118,119]. The patterns of these findings appear to be distinct from the effects of a non-specific memory impairment associated with negative symptoms often reported in other studies. 

Understanding the trajectories of SAMP and how much they are open to revision can be important for the clinical management of psychotic disorders. When integration is incomplete, “spurious autobiographical memory of psychosis” may be an important individual feature of a psychotic illness that could influence the likelihood of relapse and refractory states. 

### 2.4. Spurious Autobiographical Memory of Psychosis in Relapse

Increased “re-experience” of psychotic experiences and their associated information is often reported prior to relapse [120,121,122,123,124]. These “recalls” of psychotic experiences may depend on environmental triggers as well as the extensiveness of the stored memory traces (likely poorly integrated, as discussed above). The fact that the content of a psychotic relapse is often similar to that in previous psychotic episodes suggests the persistence of such information and the possibility of its re-activation [120,121,122,123,124]. According to observations in memory reconsolidation [125,126], each occasion of SAMP retrieval reactivates the memory for reconsolidation and strengthens the memory. Furthermore, the addition of new psychotic memory to the existing psychotic memory system makes the memory more extensive, leading to a snowballing situation as often observed in relapses [25,32]. When memory traces of psychosis are extensive, fewer additional factors (such as dopamine hypersensitivity) are required to tip the balance for the next retrieval of information from the SAMP system, leading to a higher risk of the next relapse. As the retrieval, reconsolidation, and strengthening processes perpetuate, even fewer environmental triggers or dopamine dysregulation would be required to trigger the next relapse. This prediction from the SAMP theory is consistent with the observation of a progressive increase in non-dopamine-related factors in psychosis as the number of relapses increases.

## 3. Spurious Memories in Related Conditions

### 3.1. Substance-Induced Psychosis

The use of substances such as amphetamines is associated with psychotic symptoms. Amphetamine-induced psychosis has been used as a model to understand the roles of increased monoamine activities in psychosis (e.g., by blocking dopamine transport, resulting in an increase in synaptic dopamine and noradrenaline) [127]. Although research in this area has focused on the search for biomarkers [128], the relationship between exposure to substances and relapse is striking. A community-based study has found that around half of the patients with amphetamine-induced psychosis have had at least one episode of relapse at 5-year follow-up [129]. Importantly, after the initial episode of amphetamine-induced psychosis, future recurrences of the same psychotic experiences can occur *without* further amphetamine intake. The likelihood of recurrent episodes has been suggested to be higher in those with threatening initial experiences, particularly when similar stressors are experienced [130]. These observations suggest that in substance-induced psychosis, prior memories of psychotic experiences (and consolidation during hyperdopaminergic and hyperadrenergic states) may also play a role in subsequent episodes of psychosis. 

### 3.2. Post-Traumatic Stress Disorder (PTSD)

Experiences with PTSD may have some overlap with psychosis. A recent study has found that experiences of visual phenomena in PTSD (flashbacks) and psychosis (visual hallucinations) are similar in terms of their degree of severity and components of distress [131]. Secondary psychotic symptoms have been reported in PTSD [132,133], while several studies have also suggested the experience of psychosis as a trigger for PTSD [134]. The pivotal role of memory deficits observed in PTSD [135,136] suggests that a memory framework can be helpful for furthering our understanding of the relationships between the two conditions. 

A range of memory-related phenomena can be seen in PTSD, such as persistent intrusive memories, which are typically dominated by impairments in sensory-perceptual processing of information and reduced contextual memory abilities [135,137,138]. The weakening of contextual memory in PTSD can lead to the inability to recall specific aspects of traumatic experiences, as well as reduced autobiographical memory specificity and a reduction in memory capacity [135,139,140,141]. Notably, reductions in hippocampal volumes have been found in those with prior trauma exposure, with changes in hippocampal subfields being linked to core symptoms of PTSD, such as re-experiencing intrusive memories [142]. In psychosis, disruptions in the integration of autobiographical memory into contextual schema are also observed. This may contribute to the difficulties of integration between psychotic and non-psychotic memory, leading to an increase in OGM and disrupted autobiographical continuity [104,105,118]. Interestingly, imaging studies indicated the involvement of CA1 and CA3 subfields in the hippocampus in both PTSD and psychosis, which may play critical roles in the disruption of contextual information [143,144]. 

There are, however, important phenomenological distinctions in each of these phenomena experienced in PTSD and psychosis. While individuals with PTSD tend to have more visual re-experiencing [135], individuals with psychotic disorders tend to experience auditory hallucinations (e.g., hearing voices that are related to their experiences) [145]. In addition, although experiences of flashbacks and intrusive memories in PTSD and positive symptoms in psychosis are often felt in the “present” (or “nowness” in the PTSD literature [146,147]), details of intrusive re-experiencing in PTSD are directly related to the traumatic event(s), while those in a psychotic state are not grounded in reality (particularly when interacting with delusions). 

While these studies suggest a perspective to understand PTSD symptoms in which circumscribed experiences are intensified against the background of a weakening of contextual information, the role of an anomalous increase in dopaminergic activity may also contribute to dysfunctions in higher-level contextual representations and, in turn, the manifestation of PTSD and psychotic symptoms as discussed above. Further neurobiological differentiations between the two conditions may be revealed in future studies. 

## 4. Relationship of the SAMP to Related Theories and Constructs

Earlier studies of reinforcement learning in animal models and clinical studies have reported increased learning of irrelevant associations related to striatal abnormalities with behavioral learning paradigms such as latent inhibition, learned irrelevance, and blocking [74,148,149,150,151]. Nevertheless, these models primarily addressed the impact of dopamine on the striatum and implicit memory. They have been less linked to the hippocampus and explicit memory systems (e.g., [75]).

The potential role of the hippocampus in psychosis has been proposed [69,152]. Nevertheless, these accounts have not integrated the roles of dopamine and memory formation in mediating psychosis’s trajectory. A recent account of “inefficient neural system stabilization” has proposed complex homeostatic inadequacy at the “system level of brain network connectivity”, leading to reduced efficiency and depletion of “homeostatic reserve”, which may account for some aspects of the longitudinal course of psychosis disorders [153]. This model provides a broad account of psychotic disorders and shares some elements in line with the current proposed model (such as the hyperconnectivity between brain modules in early psychotic states), although it also does not focus on the role of SAMP *per se* in impacting psychosis outcome. 

Of note, the boundary between SAMP and delusional memory, or retrospective delusion, is to be distinguished. Delusional memory refers to a phenomenon where there is a new appearance of a recalled phenomenon that is clearly delusional. The emphasis of delusional memory is on the pathological recall process, which is experienced in the present, although the content is delusional and is about a past event. In contrast, in SAMP, the memory trace has already been laid down prior to the current recall. The main pathological event in SAMP was experienced in the past, but its memory trace persisted, and the recalled event is presumed normal. It is acknowledged that, in some cases, it may be difficult to determine whether the memory representation has been laid down previously.

As far as the authors are aware, the current SAMP hypothesis uniquely proposes that the overactivity of dopamine synthesis during psychosis is expected to increase the formation of spurious autobiographical memory. These memory traces may undergo different processing in such a way that the failure to accommodate spurious memory traces may result in a distinctive cluster of memory traces, which may contribute to the likelihood of psychotic relapses. The current hypothesis also directly makes use of recent advances in the study of dopamine mediation of hippocampal plasticity underlying autobiographical memory to link clinical phenomenology and the course of psychotic disorders. 

## 5. Compatibility with Clinical Observations

The SAMP model is consistent with a number of established clinical observations that are otherwise not satisfactorily accounted for:

(1) The experiential content in relapses is similar to the original episodes. 

The similarities between the content of psychotic relapse and that of previous episodes are consistent with the view that memory plays a critical role in relapse [120,121,122,123,124]. 

(2) Trait abnormalities in semantic memory and autobiographical memory often remain in the remission state. 

The inaccessibility of psychosis memory in remission has been associated with a “sealing over” style of recovery [154]. This phenomenon is coherently accounted for by the lack of integration between psychosis memory and remission memory clusters. Subsequently, the disruption in the coherence of autobiographical memory in psychosis is evidenced in many studies [50,105,155,156,157,158].

(3) Each successive relapse becomes more difficult to treat with medication.

There is increasing evidence that after each relapse, responses to dopamine antagonist medication diminish, suggesting an increase in the cumulation of non-dopamine contributions to the psychotic episode. We proposed that one potential non-dopamine factor is the SAMP load, which increases in a cumulative manner following each relapse. The phenomenon of increasing non-responsiveness to antipsychotic treatment with relapse is consistent with the SAMP hypothesis [9,159].

(4) The duration of untreated psychosis is related to relapse and remission outcomes.

The duration of untreated psychosis—the time spent in frank psychosis before effective treatment—is an important measure associated with treatment response and the long-term clinical outcomes of psychotic disorders [160,161,162,163]. The period of active psychosis would be related to the SAMP load. The relationship between DUP and positive symptom outcomes (e.g., symptom remission, treatment response, or relapse) would therefore be consistent with the SAMP hypothesis.

The model also makes some additional observations: for instance, negative symptoms, which result in restriction in environmental exposure, or affective blunting as a result of over-medication would impede the normalization of SAMP and increase the chances of relapse.

Aside from these clinical observations, other considerations might be needed in the application of the SAMP. We use psychotic-like experiences in PTSD and suspicious/paranoid personalities as examples. 

As noted, the dopamine hypothesis posits that psychotic disorders are primarily caused by intrinsic abnormalities in the dopaminergic system [12]. In contrast, PTSD is generally considered to be a more “externally driven” condition, such that the disorder is primarily induced by exposure to a traumatic event or significant stressor and involves complex interactions between genetic, environmental, and psychological factors [136]. The SAMP perspective would anticipate that the recursive memory phenomena in PTSD as a result of traumatic exposure would involve dysregulation of the dopaminergic system. While involvement of the dopamine system has been observed in PTSD, further research would be required to further understand their roles in PTSD and how they might be similarly or differentially manifested as compared with psychotic disorders [164].

Paranoid personality disorder has also received some attention in the literature [165]. While those with this personality type are also prone to persistent paranoid ideations, their etiology may have more to do with maladaptive schema content than dopaminergic activities and may therefore be less influenced by spurious autobiographical memory. The thematic continuity of context in paranoid personalities is typically understandable without overly bizarre or spurious associations. In this context, it is important to acknowledge the role of premorbid personality in predicting relapse [166]. The assessment of premorbid personality is therefore crucial to understanding the relapse process [167]. 

## 6. Clinical Implications

The proposed SAMP theory is based on emerging basic neuroscience observations and human neuroimaging findings of dopamine and hippocampal interactions. It provides a novel account of factors that may determine treatment response to dopamine-based antipsychotic medication, as well as the phenomena of relapse and persistence of psychotic symptoms. In particular, it illustrates how a neurocognitive process could be initially driven by dopamine overactivity and evolve into a state in which dopamine no longer plays a predominant role. It suggests that a linear relationship between dopamine levels and psychotic symptoms would require review with consideration of the involvement of memory processes. 

The theory in particular highlights the importance of minimizing the duration of active psychosis for patients, which involves both the DUP period as well as the period from treatment to remission. There is also a need to distinguish between “active” psychosis and “inactive” psychosis. Active psychosis refers to states in which ”fresh” psychotic experiences are generated, whereas “inactive psychosis” refers to states in which fresh psychotic experiences are no longer generated, although existing psychotic experiences can still be expressed. Such “fresh” psychotic experiences are evidenced by the linking of current autobiographical event elements with psychotic interpretations. Research interests in active psychosis have previously been promoted with the hypothesis that brain processes during active psychosis could be neurotoxic (narrowly interpreted as producing direct deleterious effects that irreversibly damage brain structures). However, subsequent research efforts have drifted towards the assessment of DUP in first episode psychosis rather than the period of active psychosis during the first episode (which includes the DUP and the period after treatment but before response), as well as active psychosis periods throughout the longitudinal illness course. 

In the SAMP account, psychosis and memory load predict outcomes. Developing instruments and procedures for the assessment and monitoring of active psychosis in future longitudinal studies would be important. The SAMP model suggests that the overall “load”, i.e., the cumulative strength of spurious autobiographical memory of psychosis, would be a determinant factor for clinical outcomes of psychotic symptoms (relapse and refractoriness). The quantity and strength of spurious autobiographical memory of psychosis would thus depend on the cumulative periods of active psychosis (both in the prodromal phase, the untreated psychosis period, the pre-response period in the first episode of psychosis, and the active psychotic periods in subsequent relapses). 

Our review of the neurobiological processes involved in spurious autobiographical memories of psychosis revealed that there is an overlap between the brain systems and cognitive mechanisms underlying psychosis memories in psychotic disorders, amphetamine-induced psychosis, and PTSD. It would be prudent to consider this re-experiencing of memory formed under salient conditions as a spectrum phenomenon with some common core processes. The study of spurious autobiographical memory spectrum phenomena will be important for understanding nosological boundaries.

Clinically, one of the most important implications of the spurious autobiographical memory theory of psychosis is that the consolidation of spurious autobiographical memory should be minimized. This involves the reduction of the duration of untreated psychosis through early detection and intervention, as well as timely and effective treatment of active psychotic symptoms to minimize the period of active psychosis. The spurious autobiographical memory of psychosis theory would predict that prolongation of partially treated symptoms may be associated with more future re-experience of the symptoms, either in relapse or in treatment-refractory states. Likewise, relapse prevention is important. If a relapse does occur, early detection and prompt treatment are important to minimize the accumulation of psychotic memories.

The spurious autobiographical memory of psychosis theory provides a more nuanced theoretical background for facilitating better integration of psychotic experiences. Previous studies on the recovery process following a psychotic episode have identified two “recovery styles” [154,168]. In the “integration” style, the patient confronts the psychotic experience and seeks information to handle the experience; in the “sealing off” style, the patient leaves the psychotic experience unprocessed and avoids confronting the experience. A “sealing off” recovery style would inhibit the integration of psychosis memories into background autobiographical memory and facilitate their persistence as a separate memory cluster. Psychoeducation efforts for patients and caregivers should not just be the unilateral delivery of information but should also involve a dialogical process in which patients are enabled to reflect on their psychotic experiences and express their view of how they are accommodating the experiences into their autobiographical memory in a coherent manner. 

Once spurious autobiographical memory is consolidated, normalization can only occur with adequate exposure to relevant stimuli under healthy, normalized conditions. This process is expected to take longer than the formation of psychotic memories. The normalization of memory would be facilitated by exposure to a full range of healthy life experiences, especially those involving some of the contexts that were previously encountered during psychosis (for example, a setting in which suspicious thoughts were experienced). The exposure should be supported and monitored in a context where the patient feels safe. Overexposure may trigger the reactivation of psychotic memories. This process is aligned with the process of addressing “safety behavior” in cognitive behavioral therapy and with the principle of normalization in rehabilitation services. It is also suggested that the facilitation of normal memory processes may enhance normalization. Physical exercise has been demonstrated to enhance both memory function and hippocampal integrity. Cognitive rehabilitation addressing metacognition and memory may also facilitate the reintegration of memory. The importance of avoiding over-medication is also highlighted in the spurious autobiographical memory of psychosis theory.

## 7. Future Directions

The measurement of spurious autobiographical memory of psychosis load is challenging, but can be possible with careful design. Studies of SAMP may also adopt a prospective longitudinal design to provide more causal links with clinical outcomes at a future time point. Precise measurements should be developed to measure the period of active psychosis by making a distinction between positive symptoms that are being actively formed and symptoms that are the persistence of representations already formed. The individualized content of SAMP could be studied using detailed phenomenological methods, with adaptations from the field of autobiographical memory studies. Neuroimaging studies of dopamine-hippocampal interaction in a prospective longitudinal cohort of patients should reveal the potential diversity in the relationship between dopamine synthesis capacity, hippocampal functions, memory functions, and experiential information. The study of memory traces could also be approached using cognitive methods such as semantic priming, and relational memory. 

Overall, future investigations of spurious autobiographical memory of psychosis and related phenomena, as discussed in this review, require an integrative approach using methods from phenomenology, nuanced symptom quantification, and neurocognitive and brain imaging paradigms. An integrative approach to tapping memory load in autobiographical, semantic, associative, and emotion memory systems should be aimed for in future studies. 

## 8. Conclusions

We propose a new theory for understanding the course of positive symptoms in psychotic disorders based on recent findings in the neurobiology of dopamine-hippocampal interaction in the formation of memory traces. The theory accounts for a number of existing observations on the course of positive symptoms in psychotic disorders. It opens up intervention possibilities for potentially modifiable processes. The theory also provides a new paradigm in which further research questions could be asked. The model is based on recent data on the role of dopamine in the registration and consolidation of episodic autobiographical memory. Based on the established finding of increased dopamine production in psychosis, it is expected that spurious autobiographical memories will be registered and consolidated during psychotic episodes. It is also anticipated that these memories may have difficulty being integrated into pre-psychotic memory and remission memory. Unintegrated memories may persist until they are normalized by extinction processes through normalized experiences. Spurious autobiographical memories may predispose to future reactivation of psychotic-related information, experiences, and emotions. In this way, spurious autobiographical memories of psychosis lower the threshold for relapse and increase the difficulty of eliminating active psychotic symptoms. The progressive accumulation of spurious autobiographical memories of psychosis may be a mechanism that can contribute to the emergence of treatment-refractory psychotic states. The spurious autobiographical memory of psychosis theory raises the interesting and important possibility of psychological work. 

## Data Availability

Not applicable.
